# Factors Affecting Pupation Success of the Small Hive Beetle, *Aethina tumida*

**DOI:** 10.1673/031.012.11801

**Published:** 2012-10-10

**Authors:** W.G. Meikle, R. Diaz

**Affiliations:** Honey Bee Research Unit, Kika de la Garza Subtropical Agricultural Research Center, USDA-Agricultural Research Service, 2413 E. Highway 83, Weslaco, TX 78596

**Keywords:** diet, pupal stage, soil depth, temperature

## Abstract

Survivorship of larvae of the small hive beetle, *Aethina tumida* Murray (Coleoptera: Nitidulidae), was measured after they were raised on one of six diets. The effects of container shape (wide and shallow vs. narrow and deep), soil depth (0, 0.5, 1.0, 2.0, 4.0, and 8.0 cm), and temperature (28°, 32°, or 35° C) on pupation success was measured. Diet influenced larval survivorship, but did not have a strong effect on larval weight. The larvae fed only bee brood survived the shortest period of time. The larvae that were denied pupation substrate, fed only honey and pollen, and no other food or water after 20 days, had a median survivorship of 47.6 days, with a maximum of 61 days, while those fed only brood had a median survivorship of 18.2 days. Pupation substrate was essential for successful pupation, and the depth of the substrate, not its top surface area, was the crucial factor. Pupation success in narrow and deep containers was 95.6% on average, but only 12.5% in wide and shallow containers, using the same soil volume. In narrow and deep containers, most or all larvae kept in 4–8 cm of soil pupated at all temperatures, few larvae kept at 2 cm soil depth pupated, one out of 240 kept at 1.0 cm pupated, and no larvae kept at soil depths of 0 or 0.5 cm pupated.

## Introduction

The small hive beetle, *Aethina tumida* Murray (Coleoptera: Nitidulidae), is native to subSaharan Africa (Lundie 1940), and was first detected in the USA. in 1998 (Elzen et al. 1999; Arbogast et al. 2009). Adults invade honey bee colonies, where they lay eggs in crevices and on the combs. Both adults and larvae feed on pollen and honey (Lundie 1940; Meikle and Patt 2011, Meikle et al. 2012) and attack brood (Ellis and Delaplane 2008). *A. tumida* transmit diseases, including honey bee sacbrood virus (Eyer et al. 2009) and American foulbrood, *Paenibacillus larvae* (Schäfer et al. 2009), one of the most important bee diseases. In large numbers, *A. tumida* can cause hive collapse (Schmolke 1974; Neumann and Elzen 2004; Ellis and Delaplane 2008). Recently, they have been the focus of much research.

Small hive beetles are different from bee pests such as Varroa mites (*Varroa destructor* Anderson and Trueman) and wax moth (*Galleria mellonella* L.) in that late instar larvae must leave the beehive in order to pupate in the soil. Typically, the larvae bury themselves in the soil where, after several days, they metamorphose into pupae, and then emerge after a pupation period of several more days (de Guzman and Frake 2007). The total time the pupae spend in the soil, as well as pupal mortality, can vary with temperature (Meikle and Patt 2011), nutritional status (Meikle et al. 2012), and soil type and moisture content (Ellis et al. 2004). The length of time the beetle spends in the soil ranges from ∼15 days at 35° C to ∼33 days at 21° C (Meikle and Patt 2011). Another potential factor influencing pupation is diet. *A. tumida* have a broad food range, and have been raised on fresh and rotten apples, oranges, cantaloupe and grapes (Ellis et al. 2002; Arbogast et al. 2009), pollen and honey (Lundie 1940; Ellis et al. 2002), and bee brood (de Guzman and Frake 2007). Ellis et al. (2002) reported significant effects of diet on pupation success and adult longevity.

The movement of mature larvae from beehive to soil presents an opportunity for beekeepers to apply control measures outside the hive. For example, entomopathogenic nematodes and fungi have been found to be effective biological control agents when applied as a soil treatment against the soil-dwelling stages of other pests, including the viburnum leaf beetle, *Pyrrhalta viburni* (Paykull), (Weston and Desurmont 2007), and pollen beetles, *Meligethes* spp., (Nielsen and Philipsen 2005; Husberg and Hokkanen 2001). Entomopathogenic nematodes are known to attack *A. tumida* (Cabanillas and Elzen 2006).

Knowledge of the minimum soil depth necessary for pupation, and how that depth might be influenced by temperature or diet, would be useful in designing traps and implementing control strategies. Based on previous experience with *A. tumida* (see Meikle and Patt 2011), we hypothesized that larvae would not pupate in the absence of soil, and that diet, if restricted to foods typically found in bee hives (i.e. pollen, bee brood, artificial pollen and honey), would have a limited effect on larval survivorship. If larvae would not pupate in the absence of soil, then we hypothesized that there must be a minimum depth of soil needed for pupatation. The first hypothesis was addressed by feeding larvae different diets, and weighing and observing the larvae until they either pupated or died. Next, experiments were conducted to determine how soil distribution (i.e., in large, shallow containers, or narrow, deep containers) and/or soil depth (i.e., 0.5, 1.0, 2.0, 4.0, or 8.0 cm deep) would influence *A. tumida* pupation success. The work was done at three temperatures, 28°, 32°, and 35° C, to evaluate the interaction of temperature with soil depth. The baseline temperature of 28° C was chosen because *A. tumida* pupate relatively quickly at that temperature (usually about 17 days) with high survivorship (> 90%) (Meikle and Patt 2011). The effects of high-temperatures likely to be encountered in subtropical soils were of particular interest in this study. The work here is intended to contribute to the knowledge of *A. tumida* ecology outside the hive.

## Materials and Methods

### Insect rearing.

For each batch of *A. tumida* larvae, 40–50 adult beetles from lab cultures (founded with beetles caught in the vicinity of Weslaco, TX, USA) were placed in a 1 L (8.5 cm ×30 cm × 21 cm) plastic oviposition chamber containing ∼230 g standard pollen patty (see below), and a 10 × 10 cm piece of brood comb spread with 10 mL of honey. Beetles were kept in the chamber at 32° C for 3 days, during which they laid eggs. After eggs were laid, the adults were removed, and the chamber was incubated at 32° C. This procedure yielded ∼5000 mature, wandering larvae in about 14 days in each batch. These larvae were transferred to a 2 L jar filled with moist, sandy soil (moisture content 5%) to pupate, and were kept at 22–26° C until adults emerged 3–4 weeks later.

### Survivorship with different diets.

Different diets, containing ingredients likely to be found in a beehive, were prepared (Table 1). Standard protein patty represents a diet similar to what beekeepers may add to hives, and included the artificial pollen Bee Pro (Mann Lake Ltd, http://www.mannlakeltd.com/). For the experiment on the role of diet in larval survivorship without pupation substrate, eggs were collected by placing oviposition slides similar to those described by de Guzman and Frake (2007) in an oviposition chamber. Oviposition slides were constructed by first affixing one glass cover slip (18 ×18 mm) at each end of a standard glass microscope slide (75 × 25 × 1mm), using Superglue (Henkel Corp., http://www.henkel.com), and then affixing a second slide on top, the oviposition site being the narrow space between the slides. Eggs laid in oviposition slides were counted using a dissecting microscope. A 2 cm^2^ hole was cut in the lid of each vial, and a piece of nylon gauze was glued over the hole to provide ventilation. Larval feeding chambers consisted of a single oviposition slide, with eggs and 2 g of diet placed in a 120 mL polypropylene specimen vial with a screw-cap (Kendall vials, Covidien, http://www.covidien.com). Larvae hatched from eggs in the slide, and were monitored as they fed. Diet was supplemented as needed until it was evident that feeding had stopped. Any leftover food was removed and discarded on day 20. Larvae were raised at 32° C on 6 diets: SPP, HP, HP0. 1, HP1.0, HPB, and brood alone. Each diet treatment had seven replicates, except for the brood alone diet, which had eight. Larvae were counted and weighed on an electronic balance (OHaus Corp., http://www.ohaus.com) at least once a week until all had died. Average survival time per vial was used in the statistical analysis.

**Table 1.**
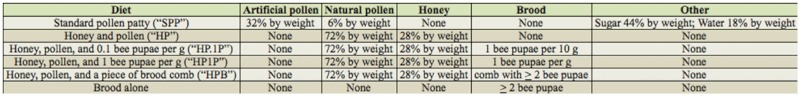
A list of the diets used in this study and their ingredients.

### The effects of soil volume and container shape on pupation.

Two kinds of containers were prepared: transparent Petri dishes (14.0 cm diameter and 2 cm depth), and transparent plastic tubes (4.1 cm diameter and 20 cm depth). The plastic tubes were prepared by cutting plastic tubing (intended for protecting fluorescent lighting) into 20 cm long sections. Plastic sheeting was glued onto the bottoms of the tubes in order to hold soil and prevent water loss. Two kg of sandy loam soil was prepared by sifting and adding water until the soil was moist but not wet. Soil moisture content was measured by weighing six 1 g samples in small plastic containers, drying the samples for 2 weeks in a crystallizing dish containing silica gel, and then re-weighing. Soil was divided into 40 mL and 80 mL samples, and one soil sample was placed in each container. In the Petri dishes, this procedure resulted in a depth of 0.26 cm and 0.52 cm for the 40 mL and 80 mL samples, respectively. In the plastic tubes, this resulted in depths of 3.03 cm and 6.06 cm for the 40 mL and 80 mL samples, respectively. Four replicates were prepared for each container and soil volume treatment. Ten two-week-old larvae (raised on SPP, brood comb, and honey) were placed in each container. Petri dishes were closed with Parafilm. The tops of the tubes were closed with Parafilm on top of a piece of nylon gauze, held in place with a rubber band, to prevent both moisture and beetle escape, but to still allow some gas exchange. Soil moisture was visually monitored during the study, and some water was added to experimental units as required to maintain the same soil moisture content. Emergence from the pupation tubes was monitored daily until no more beetles emerged (about 4 weeks). Containers with unaccounted-for beetles were sifted, and each insect was recorded as living or dead, and as larva, pupa, or adult. The experiment was conducted twice at 28° C.

### Effects of soil depth and temperature.

The same tubes used in the experiments described above were prepared with five depths of sandy loam soil: 0.5, 1.0, 2.0, 4.0, and 8.0 cm, as well as a control treatment with no soil. Four replicates were prepared for each depth treatment. Beetle emergence was recorded as above. Experiments were conducted twice at 28°, 32°, and 35° C in controlled-temperature cabinets. As above, soil moisture content was monitored, and small amounts of water added as needed.

### Statistics.

Larval longevity was evaluated using Kaplan Meier log rank analysis (SigmaPlot 11.0) with pairwise comparisons. Emergence data were analyzed using SAS software (SAS Institute, Inc., http://www.sas.com). In all analyses, the experimental units were specimen vials, and average values per vial were used in the statistical analyses. ANOVA analyses (α = 0.05) were conducted for linear mixed models using PROC MIXED (Littell et al. 1996), with experiment number as the random effect. Percentage of larvae surviving and percentage reaching the adult stage were arcsine square-root transformed, as is recommended for percentages that cover a large range of values (Steel and Torrie 1960), and untransformed values were presented graphically. For PROC MIXED analyses, degrees of freedom were calculated using the Satterthwaite method, type III sums of squares were used where applicable, and residual plots were assessed visually for variance homogeneity. Post hoc contrasts of the least squares means differences were conducted for all significant factors, using the Bonferroni adjustment for the t-value probability. Significant interaction effects were evaluated using test-of-effect slices.

## Results

### Survivorship with different diets.

**Figure 1.**
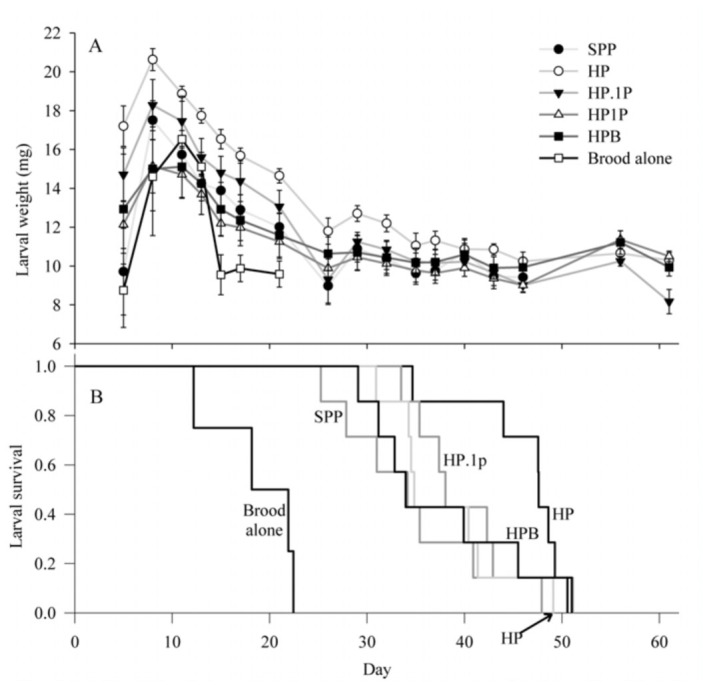
Larval weight changes and longevity for *Aethina tumida* larvae in an experiment with six diet treatments and no pupation substrate. A) weight changes over time; B) Kaplan Meier analysis of survivorship times. SPP: standard pollen patty (see text for details); HP: honey and pollen only; HPO. 1 : honey, pollen and 0.1 bee pupae per g diet; HP1 .0: honey, pollen and 1 bee pupa per g diet. High quality figures are available online.

Larvae denied a pupation substrate lived as long as 61 days; no larvae pupated, but most eventually became prepupae. No larvae fed brood alone survived more than 21 days. Diets were ranked as follows (median survivorship in days): HPP (47.6), HPP+. 1 (38.0), HPP+1 (34.8), SPP (34.1), HPB (34.0), and brood alone (18.2). The Kaplan Meier analysis showed that diet was a significant factor (log rank statistic = 60.72; *p* < 0.001), but only pairwise comparisons involving brood alone were significant (unadjusted *p* = 0.0004 for all) (Figure 1). A repeated measures analysis of larval weight including all six diet treatments, conducted using data up to day 21, showed a significant interaction of time and diet (F = 3.21; df = 30, 185; *P* < 0.0001), but not diet alone (*p* = 0.16). Removing the brood treatment allowed the analysis to continue until day 46, when all larvae in the SPP group had perished. Diet had a significant effect in that case (F = 3.17; df = 4, 36; *p* = 0.0250), but only one post hoc contrast in that analysis was significant, showing that larvae raised on HP were significantly larger than those raised on HP1.0(p = 0.0496).

### The effects of soil volume and container shape on pupation.

Soil moisture content was 6–8% at the start of the study, and was maintained at a minimum of that level throughout the study. Regardless of volume, larvae placed in tubes had a much higher success rate than those placed in dishes (F = 228.81; df = 1, 27; *P* < 0.0001). Larvae placed in 80 mL of soil pupated better than those placed in 40 mL soil, regardless of container type (F = 5.51; df = 1, 27; *p* = 0.0265) but the container type and volume interaction was not significant (*p =* 0.15). Successful pupation in the tubes was high, with an average of 92.5 ± 7.5% and 98.8 ± 1.3% for 40 mL and 80 mL of soil in tubes, respectively. In dishes, successful pupation was low, with an average of 3.8 ± 1.3% and 21.3 ± 8.8% for 40 mL and 80 mL of soil, respectively. Beetle development in dishes appeared delayed. After three weeks, all larvae had either pupated or died in the tubes, but 72.5% of the original larvae in the Petri dishes were still live larvae, 14.4% were dead larvae, and 10.0% were pupae that later eclosed as adults.

### Effects of soil depth and temperature.

Soil depth (F = 633.50; df = 5, 126; *p* < 0.0001), temperature (F = 6.77; df = 2, 126; *p* = 0.0016), and their interaction (F = 3.03; df = 10, 126; *p* = 0.0018) all had a significant effect on beetle survivorship. Beetles at 8 cm depth had higher survivorship than those at 4 cm depth (*p* = 0.0117), and a significantly greater proportion of those two groups survived than did beetles at all the other depths (*p* < 0.0001 for all contrasts). Beetles kept at 32° C did significantly better than those at 35° C, but no other contrasts for temperature were significant. Tests of effect slices showed a significant interaction with temperature at 2 cm and 4 cm depth.

**Figure 2.**
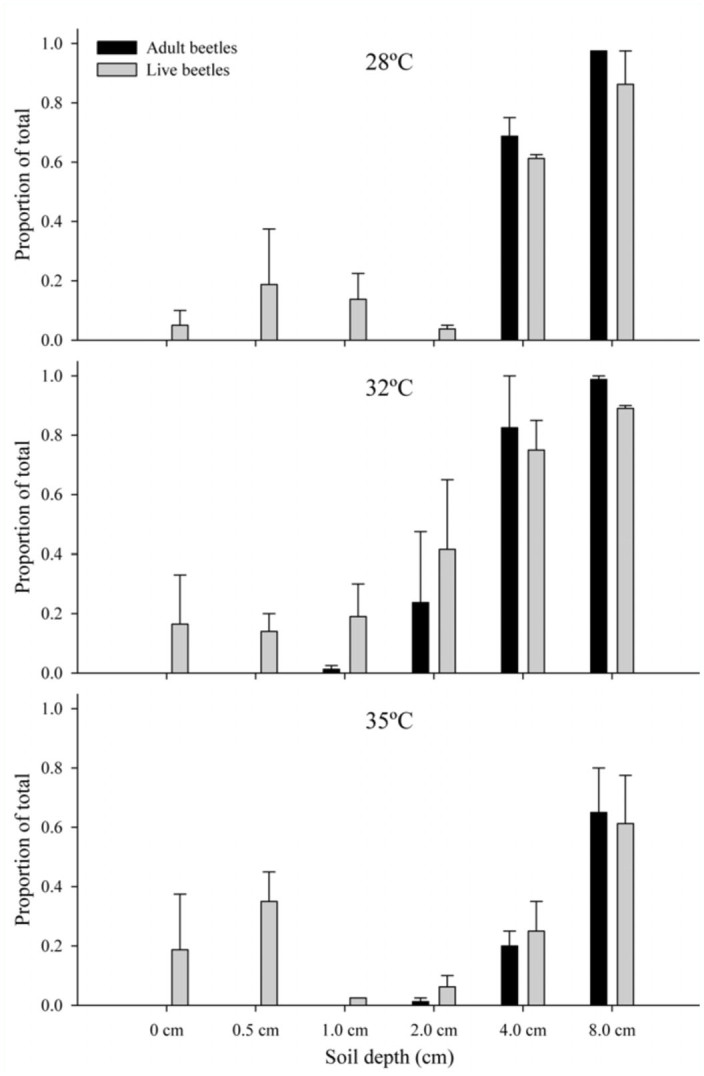
Proportion *Aethina tumida* larvae that survived or successfully pupated to the adult strage at six soil depths and over 3 temperatures. Bars show experimental averages ± SE. High quality figures are available online.

Soil depth had a strong effect on the proportion of larvae that successfully pupated into adults (F = 160.34; df = 5, 125; *p* < 0.0001) (Figure 2). A single beetle (out of 240 larvae at that depth) pupated successfully at 1.0 cm soil depth. Temperature did not have an effect (*p* = 0.18), but the interaction of depth and temperature did (F = 6.40; df = 10, 125; *p* < 0.0001). Post hoc contrasts showed that all depths 2.0 cm and shallower had a significantly lower proportion of successful pupation than those 4.0 cm and deeper.

## Discussion

*A. tumida* is unusual among honey bee pests in that it must exit the hive to complete its life cycle. While the beetle larvae inflict their damage before leaving the hive, their leaving the hive offers a window for a non-invasive control strategy. The experiments in this study were intended to help clarify aspects of beetle nutrition and ecology with respect to the pupal stage.

Beetle larvae kept in rearing vials without any pupation substrate lived up to 61 days, but never pupated, indicating that at least some pupation substrate is necessary for development. Larvae did not eat during that period, and may have been too weak to complete development for some time before death. But, the larvae were able to survive long periods without food, water, or substrate, and they could potentially travel long distances to seek substrate. Larvae fed diet with at least some pollen lived the longest. Larvae fed only brood grew to about the same weight as larvae fed other diets, but survived a maximum of only 21 days. Survivorship varied among diets with pollen, but the effects were comparatively minor. One of the first researchers to study *A. tumida*, Lundie (1940) measured the duration of *A. tumida* life stages using only pollen and honey as diet, and considered bee larvae and eggs minor parts of the *A. tumida* diet.

The experiments involving different container shapes and soil volumes were designed specifically to address the question of whether the distribution of the pupation substrate (i.e., with a large or small surface area, and whether shallow or deep) was important to pupation success. The Petri dishes had surface areas of 153.9 cm^2^, which was 11.7 times larger than that of the tubes (13.2 cm^2^). So, given a constant soil volume, the tubes had soil 11.7 times deeper (3.03 cm and 6.06 cm depth for 40 mL and 80 mL soil, respectively, compared to 0.26 cm and 0.52 cm depth) than that for the corresponding volume of Petri dish. Soil volume was a significant factor, with pupation success across container types of 48.1% in 40 mL of soil and 60.0% in 80 mL, but container type had a much stronger effect. Only 12.5% of the beetles emerged in the Petri dishes across experiments, while emergence in the tubes was 95.6%, regardless of soil volume. Clearly vertical space, in the form of soil depth, rather than horizontal space, in the form of top surface area, played the larger role in pupation success.

Substrate depth and temperature were examined together in further experiments. At the end of these experiments, each individual was classified into one of three groups: adult (completed pupation), larva (alive but not pupated), or dead. No successful pupation was observed in soil 0.5 cm deep, although five emerged at about that depth in Petri dishes in the study described above. At 0.5 cm soil depth, the Petri dishes had far more soil volume (80 mL) than the tubes (6.6 mL). Only one adult emerged out of 240 larvae (across all temperatures) at 1.0 cm soil depth. While some larvae pupated at 2.0 cm depth, success was significantly improved with at least 3 cm of soil, as observed in the container type study. Clearly, 35° C is too warm for proper pupation, regardless of soil depth. Meikle and Patt (2011) also observed low pupation success at 35° C. Average temperatures at 10 cm depth range from 24° C on the Gulf Coast of the USA to ∼8° C on the US-Canadian border, but the soil temperatures are increasing (Hu and Song 2003).

The results indicate minimum pupation depth, rather than preferred depth. During the course of these experiments, it was observed that while larvae often dug tunnels throughout the pupation tube no matter what the depth was (in laboratory beetle cultures, deeper pupation containers are used and similar tunnels observed at least 30 cm deep), they could be found forming pupation chambers close to the surface, even < 1 cm deep. It is likely that, given the opportunity, larvae could dig much deeper, although that may be a different issue from their preferred pupation depth. Soil type is known to be an important factor in pupation success (Ellis et al. 2004), but soil type was not examined in this study. The sandy loam used as a pupation substrate was found to result in high rates of pupation success in previous experiments (see Meikle and Patt 2011), and was for that reason used in this study. Ellis et al. (2004) showed that soil moisture content played an important role, and found little or no successful pupation in dry soil compared to moist soil. In our study, we maintained a generally constant moisture content by inspecting the soil and adding moisture if needed, so low soil moisture content would generally have played a small role. However, experimental units with very small amounts of soil may have experienced more variability than others, and this may partly explain the low pupation success, for example, in tubes with 1.0 cm depth compared to dishes with 0.52 cm depth. Although the soil was shallower in the dishes, they would have contained more soil than the tubes and thus soil moisture content would likely have fluctuated less.

These results do have implications on possible control strategies based on the pupal stage. Simply preventing larvae from accessing pupation substrate would eventually kill most or all of the wandering larvae. The soil depth results indicate that larvae will likely burrow at least 2 cm before pupating. To prevent *A. tumida* pupation where bee hive parts are stored and honey is extracted, it would be necessary to make sure that any potential pupation substrate, such as soil but possibly other materials, does not build up into layers or drifts deeper than that. In the field, these results indicate that few larvae will pupate in the top 2 cm of soil, so control through physical disturbance would need to take that into account. Clearly, larvae need some sort of pupation site, but whether larvae need a particulate substrate like soil for pupation, or whether other sites are acceptable, remains to be explored.
